# A call for clinical trial globalization in Alzheimer’s disease and related dementia

**DOI:** 10.1002/alz.12995

**Published:** 2023-02-25

**Authors:** Jorge J. Llibre-Guerra, Anika Heavener, Sonia Maria Dozzi Brucki, Juan Pablo Díaz Marante, Maritza Pintado-Caipa, Yaohua Chen, María Isabel Behrens, Angela Hardi, Arianna Admirall-Sanchez, Rufus Akinyemi, Suvarna Alladi, Karen A. Dorsman, Ana M. Rodriguez-Salgado, Joel Solorzano, Ganesh M. Babulal, Diversity and Disparity Professional Interest Area Increasing Sustained Diversity in Clinical Trials Working Group

**Affiliations:** 1 Department of Neurology, Washington University, School of Medicine, St. Louis, Missouri, USA; 2 Dominantly Inherited Alzheimer’s Network Trial Unit, St. Louis, Missouri, USA; 3 Institute of Public Health, Washington University, St. Louis, Missouri, USA; 4 Department of Global Health and Social Medicine, Harvard Medical School, St. Louis, Missouri, USA; 5 Cognitive and Behavioral Neurology Unit, Department of Neurology, University of Sao Paulo, São Paulo, Brazil; 6 Department of Primary Care, Espirutu Santo, Brazil; 7 Instituto Peruano de Neurociencias, Lima, Peru; 8 Department of Geriatrics, Lille Neurosciences & Cognition, University of Lille, Lille, France; 9 Departamento de Neurología y Neurocirugía, Hospital Clínico Universidad de Chile, Independencia, Santiago, Chile; 10 Becker Medical Library, Washington University School of Medicine, St. Louis, Missouri, USA; 11 Centre of Public Health, Trinity College Dublin, Dublin, Ireland; 12 Centre for Genomic and Precision Medicine, College of Medicine, University of Ibadan, Ibadan, Nigeria; 13 National Institute of Mental Health and Neuroscience, Bangalore, India; 14 University of Texas Southwestern Medical Center, Dallas, Texas, USA; 15 Global Brain Health Institute, San Francisco, California, USA; 16 Department of Medicine, Hospital Antonio Luaces Iralola, Ciego de Avila, Cuba; 17 Department of Clinical Research and Leadership, The George Washington University School of Medicine and Health Sciences, Washington, DC, USA; 18 Department of Psychology, University of Johannesburg, Johannesburg, South Africa

## Abstract

**Background:**

The burden of Alzheimer’s disease and related dementia (ADRD) is projected to disproportionally impact low-middle-income countries (LMICs). However, there is a systematic under-representation of LMICs in ADRD clinical trial platforms.

**Methods:**

We aimed to determine the global distribution of ADRD clinical trials and identify existing barriers for conducting clinical trials in LMICs. Primary data sources to identify trial distribution in LMICs included ClinicalTrials.gov and the International Trials Registry Platform. An additional systematic review and expert consensus interviews were conducted to identify barriers for conducting clinical trials in LMICs.

**Findings:**

Among 1237 disease-modifying therapies tested in ADRD clinical trials, only 11.6% have been or are conducted in emerging economies (upper-middle income [9.6%] and low-middle income [2.0%]). We identified several limitations for trial implementation including a lack of financial resources, low industry presence, regulatory obstacles, and operational barriers

**Interpretation:**

Although LMICs bear the greatest burden of ADRD globally, substantial development of clinical trial platforms to address this inequity and health disparity is lacking.

## 1 | INTRODUCTION

In the absence of clinically meaningful disease-modifying treatments (DMTs), the number of adults with dementia worldwide is projected to more than triple.^1,2^As a consequence, there is an urgent need for therapies that could delay or disrupt the progression to dementia. Currently there are several therapeutic agents in more than 1000 clinical trials for Alzheimer’s disease and related dementias (ADRD); several of those concluded or are close to finishing their Phase 3.^3,4^ Despite the increase in the number of clinical trials over the past 20 years in ADRD, ethnoracially diverse individuals remain historically and systematically under-represented.^5–8^ Furthermore, globalization of ADRD clinical trials has become a pressing need as 68% of the people living with ADRD will reside in low-middle-income countries (LMICs) by 2025.^9,10^

Globalization of ADRD trials is a seminal strategy to address context-specific questions related to biological and non-biological variations that exist across populations. Cross-population differences may influence treatment response and frequency of adverse events in response to investigational products.^11^ In addition, the results and applicability of trials conducted in high-income countries (HICs) may not be immediately applicable to LMICs. Reasons include unequal access to health care and differences in stage at disease diagnosis, structural and social determinants of health that deeply influence the affordability of the new interventions, and limitations in resources needed to administer and monitor the new interventions (e.g., additional expertise and structural resources).^12–14^ In addition, current ADRD clinical trials are typically designed for populations in HICs and use resource-intensive measures such as multimodal imaging biomarkers and cognitive tests tailored for predominantly white, affluent, educated, and English-speaking populations.

These limitations demand a call for improvement in the recruitment, enrollment, and retention of diverse populations in dementia clinical trials to increase access and reduce health disparities.^7,15^ There is a need for a more thoughtful approach and strategies to expand the inclusion of a diverse population in ADRD clinical trials in LMICs. Democratization of clinical trials (CT) will ensure that potential treatments are safe, effective, accessible, and equitable.^16^ Because ADRD remains a prominent issue for global health and precision medicine, we sought to perform a comprehensive assessment of the international distribution of ADRD DMTs clinical trials, identify existing barriers for conducting ADRD clinical trials in LMICs, and provide recommendations to expand ADRD clinical trials to LMICs.

## 2 | METHODS

The study design included two search strategies. The first step aimed to establish the global distribution of dementia clinical trials and the second step aimed to identify existing barriers for conducting ADRD clinical trials in LMICs. The search strategies were developed with assistance from a research committee formed by a medical librarian (A.H.), ADRD researchers from multiple regions (Africa, Asia-Pacific, South America, Central America and the Caribbean, North America, and Europe), clinical trialists, and other stakeholders with expertise in clinical trials. The research committee and members of the working group on Increasing Sustained Diversity in Clinical Trials provided feedback and guidance on the proposed search strategies, selection criteria, and data analytic approaches. Details about the working group goals, interactions, and membership are provided in Supplemental Material 1.

### 2.1 | Global distribution of dementia clinical trials

**FIGURE 1:**
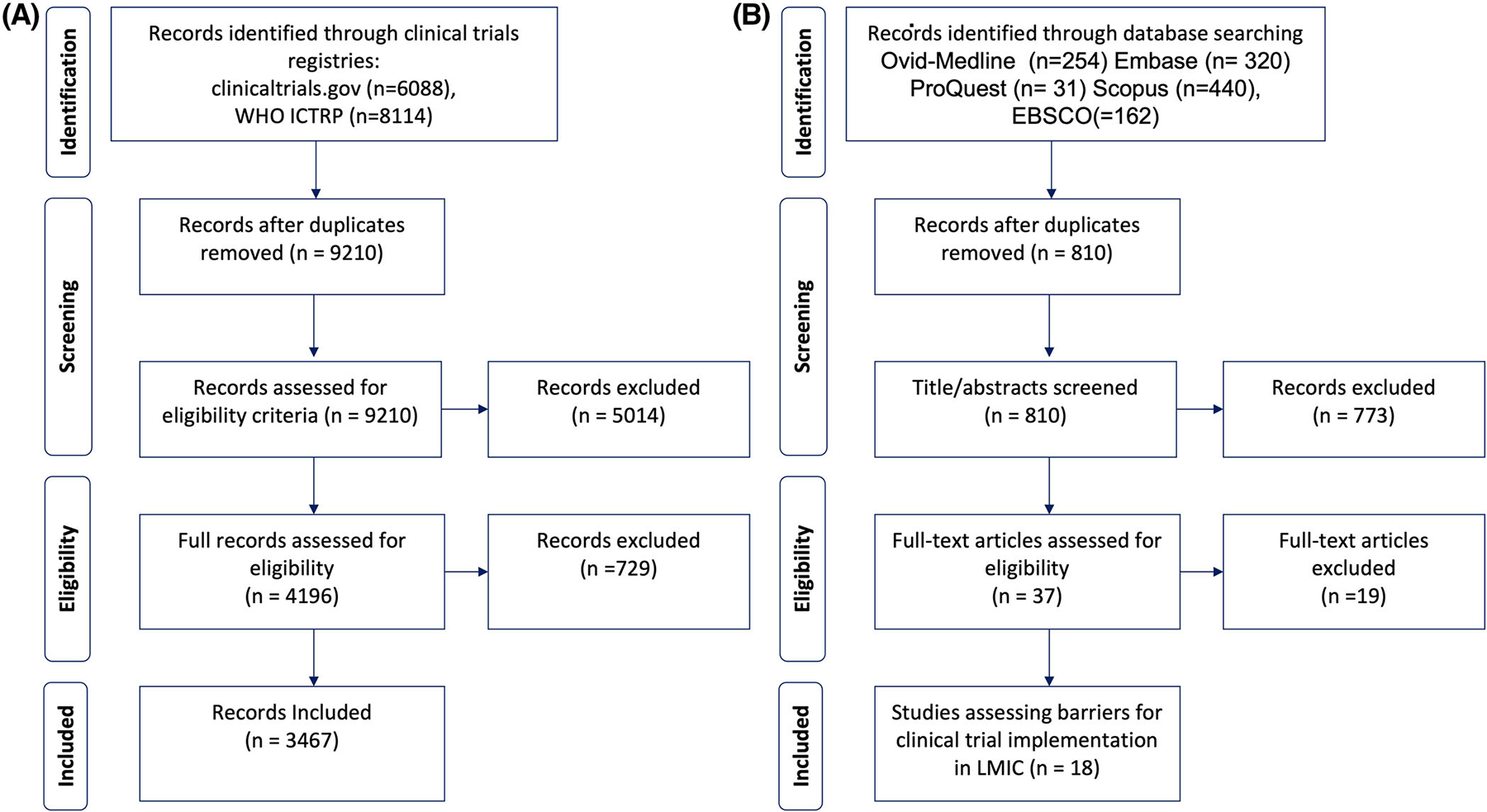
PRISMA 2009 flow diagram for study selection. Panel A. Indicates sources and steps to determine global distribution of dementia clinical trials. Panel B. Indicates sources and steps to identifying barriers for conducting dementia clinical trials in LMICs.

To determine the global distribution of dementia clinical trials, the primary data sources included ClinicalTrials.gov^17^ and the World Health Organization’s (WHO) established International Clinical Trials Registry Platform (WHO-ICTRP).^18^ Both registries are regarded as valid data sources for the study of clinical trials and are considered the largest and most comprehensive clinical trial registry worldwide with the highest compliance rates for trial registration.^19–21^ The search strategy in both trial registries used the terms: “Alzheimer,” “frontotemporal dementia,” “Lewy body disease,” “vascular dementia,” and “dementia.” We included all trials of DMTs in Phases 1, 2, and 3. We excluded all non-pharmacologic therapeutic approaches such as devices, cognitive therapies, caregiver interventions, supplements, and biomarker validation trials. Because one clinical trial may be registered in multiple countries, we obtained data on individual trials per country/region to better represent the volume of clinical research activity in different countries. Due to the potential of under-reporting of ongoing CT in LMICs, each regional co-author collected information on ongoing regional trials not reported in ClinicalTrials.gov or in WHO-ICTRP. This search yielded 14,202 studies as of January 2022 (Figure 1, Panel A). Duplicate trials (n=4992, trials included in both registries) were accurately identified and removed for a total of 3467 clinical trial studies (1237 unique DMTs).

The final data set included trial title and number, beginning date, duration, planned enrollment number, primary funding source, study phase, and country. The funding source was categorized as government-sponsored (e.g., “National Institutes of Health” or “Other Government Agency”), industry-sponsored (“Pharmaceutical companies”), Research/Academic centers (“Health care centers/University”), or a combination of the previous categories. The clinical study phase was based on the U.S. Food and Drug Administration’s (FDA’s) classification system (Phase 1 was defined as “conducted with healthy volunteers and emphasize safety and dose”; Phase 2 was defined as “preliminary data on efficacy and side effects”; Phase 3 was defined as “information about safety and effectiveness by studying different populations and different dosages”). Some trials are presented as 1/2 or 2/3 in the database, and we adopted that nomenclature in the review.

### 2.2 | Identifying barriers for conducting dementia clinical trials in LMICs

To identify existing barriers and provide recommendations for conducting ADRD clinical trials in LMICs, we conducted a systematic review search and expert consensus.

Details about the systematic review search are provided in Supplemental Material 2. In summary, the published literature was searched using strategies designed by a medical librarian (A.H.) for the concepts of research or clinical trials AND barriers, with related synonyms. These strategies were created using a combination of controlled vocabulary terms and keywords and were executed in Embase.com, Ovid-Medline All, EBSCO Global Health, Scopus, and ProQuest Dissertations and Theses Global from database inception. No language or date limits were used. All database searches were completed on March 2, 2022. A total of 1207 results were retrieved from the database literature search and exported to Endnote. Duplicate citations (n=397) were accurately identified and removed using a technique described by Bramer et al.^22^ After removing all duplicates, 810 unique citations remained for analysis and were screened for appropriateness against the inclusion and exclusion criteria. Inclusion criteria required that studies (1) reported on barriers/challenges for conducting research, specifically clinical trials within developing countries; and (2) were empirical, peer-reviewed research studies (commentaries, editorials, and literature reviews were excluded).

After the abstract screening phase (led by co-authors S.D.B., M.P.C., K.D., Y.C.), studies that met the inclusion criteria (n=37) underwent full-text assessment for eligibility (second screening stage) and were selected based on their relevance. Eighteen (18) peer-reviewed publications were selected for the final analysis (Figure 1, Panel B). The quality of each selected study was assessed according to a six-point scale (design, sample size, data collection, analysis, and report). Due to the limited number of reports, the purpose of the quality assessment was to determine the overall contribution and relevance of each report for the review and not to establish thresholds for study exclusion. A group composed of three of the authors (M.P., M.I.B., and J.J.L.G.) summarized the literature that was found per region. Collaborative regional studies were reviewed during consensus meetings. From each research study, information on the region/country, economic development, and barriers/limitations for trial implementation was extracted.^23^ This study was reported according to the Preferred Reporting Items for Systematic Review and Meta-Analysis (PRISMA) guidelines.^24^

To acquire additional perspectives on conducting clinical trials in LMICs, we complemented a 1:1 qualitative, semi-structured interview with stakeholders in ADRD research and clinical trials (led by co-author A.H.). Sampling procedures, inclusion/exclusion criteria, and data collection methods have been published elsewhere.^25^ In summary, study participants were required to be: (1) active in ADRD research, (2) responsible for enabling a particular aspect of ADRD clinical trials (ranging from clinical trial design to patient recruitment to regulatory approvals), and (3) had exposure to supporting ADRD trials in LMICs in the past 5 years. The sample size included 19 study participants representing four key stakeholders: (1) for-profit industry executives (n=7), (2) researchers or key opinion leaders (n=7), (3) government or regulatory leaders (n=1), and (4) members of non-profit organizations and patient advocacy groups (n=4). Study participants who work professionally in more than one country/region were asked to share their experience for each location. Core topics during the 1:1 interviews included: (1) identification and selection of trial sites, (2) resources and stakeholder identification, and (3) development of partnership and multi-stakeholder collaborations.

Evidence obtained from the systematic review and 1:1 semi-structured interview was synthesized using thematic analysis/synthesis,^26^ where important or recurrent themes were identified by tabulating key information across studies. Identified barriers in both studies were classified as operational, regulatory, and resource-related (e.g., human capital, equipment, financial) by a research committee. Recommendations for trial implementation in LMICs were provided by expert consensus, which included representatives from multiple LMICs (local dementia experts, clinical researchers), clinical trialists, and stakeholders with expertise in clinical trials.

### 2.3 | Statistical analyses

Data analysis included the number of clinical trials per country, regional distribution, funding source, study phase, and the number of clinical trial growth rates by region. We classified each country’s economic development status according to the 2021 World Bank development report (https://wdr2021.worldbank.org/the-report). The number of clinical trials per country and region is presented in absolute values and relative to dementia prevalence. Dementia estimates for each country were obtained from the 2019 Global Burden of Disease Study.^27^ All calculations were performed using R (v.1.2.5033) statistical computing program (https://www.R-project.org/).

### 2.4 | Funding source

No funding was provided for this study. Authors’ funding source is provided in the disclosure section. Authors’ funding source had no role in the design and conduct of the study; collection, management, analysis, and interpretation of the data; preparation, review, or approval of the manuscript; and decision to submit the manuscript for publication.

## 3 | RESULTS

### 3.1 | Global distribution of dementia clinical trials

#### 3.1.1 | Clinical trials regional distribution by economic development status

**FIGURE 2:**
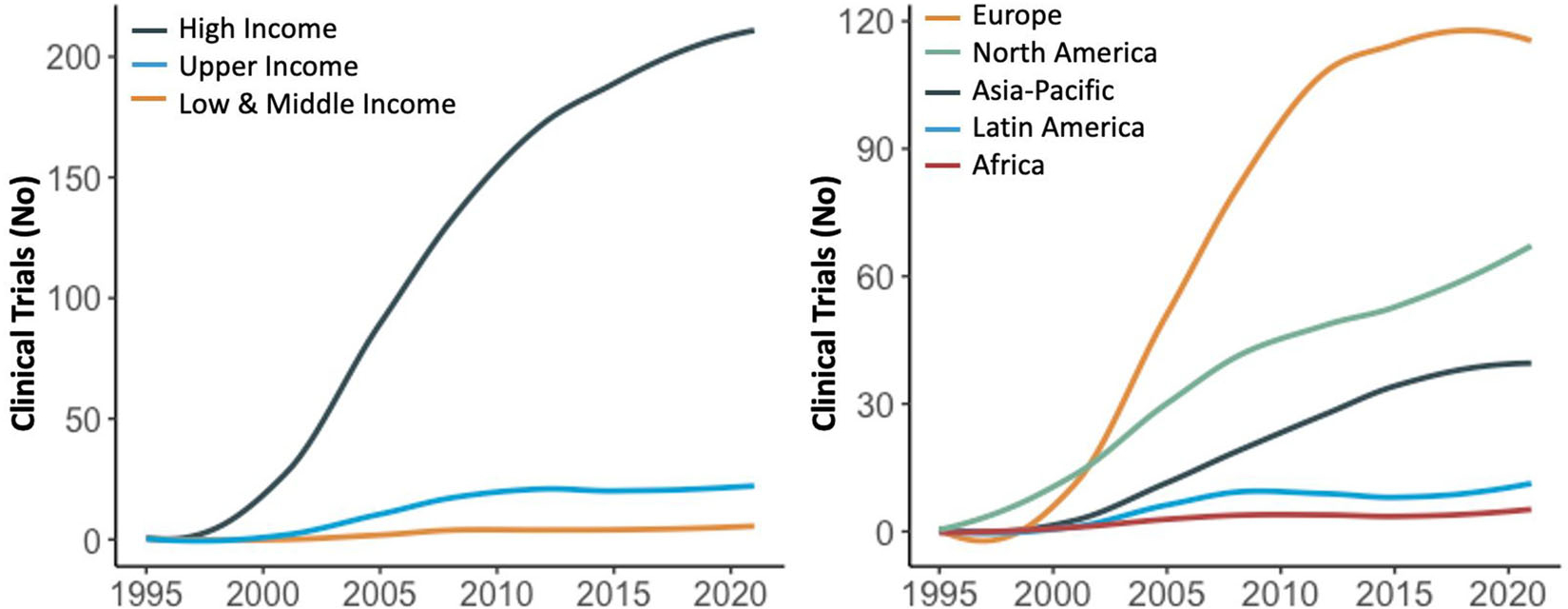
Total number of registered clinical trial growth by economic development status and region, 2000–2021. Represent the absolute trial number without taking into consideration the number of countries represented in each category. North America included the United States and Canada (Mexico is included as part of Latin America).

Over the 21-year study period, 1237 unique ADRD disease modifying therapies (ADRD-DMTs) have been tested in 74 countries, for a total of 3467 clinical trials. Among those, 3065 clinical trials (88.4%) have been conducted in HICs and 405 (11.6%) in emerging economies (upper-middle income [n=333, 9.6%], and low-middle income [n=68, 2.0%]). Over the two-decade period, HIC economies operated 97.0% of all Phase 1 clinical trials. The distribution of clinical trials by phase and economic development status are shown in Table 1. Finally, the global number of ADRD clinical trials increased from 12 to 241 (on average 15.3% growth rate per year) in HICs while the number among emerging economies countries increased from 1 to 45 (on average 19.9% growth rate per year) (Figure 2).

**FIGURE 3:**
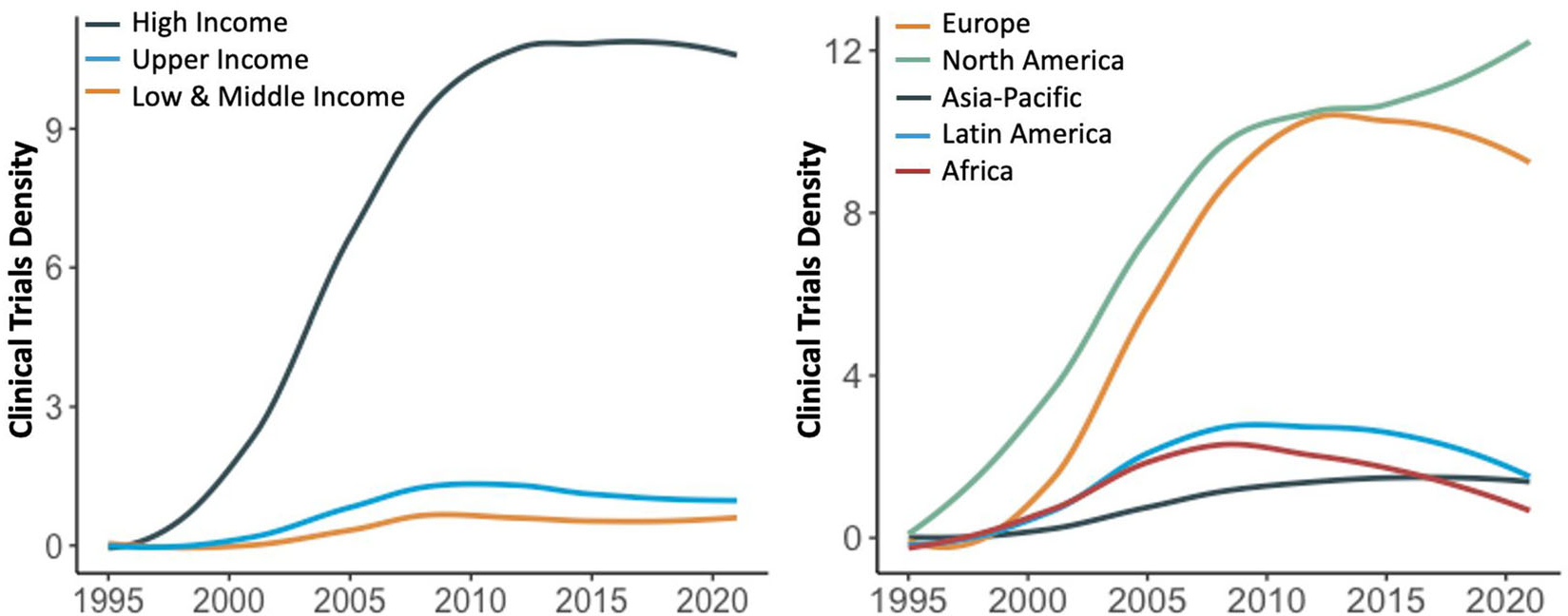
Clinical trial density by region relative to the number of people with dementia, 2000–2021. Trial site year density was the number of registered clinical trial per years divided by regional dementia prevalence. Dementia estimates for each country were obtained from the 2019 Global Burden of Disease Study.^27^

The annual average clinical trial density by economic development status, which accounts for people living with dementia (per million), is shown in Figure 3 and Table S1. HICs had the highest annual trial density growth, ranging from 0.2 in 2000 to 12.4 in 2021. Upper-middle-income countries and lower-middle-income countries had the lowest annual trial density growth (0.1 in 2000 to 2.9 in 2021) despite the relatively large population living with dementia.

#### 3.1.2 | Clinical trials regional distribution

The regional distribution of clinical trials by study phase are shown in Table 2. Overall, 78.8% of all clinical trials were conducted in Europe (n=2052, 51.4%) and North America (n=1239, 27.4%). When stratifying clinical trial regional distribution by study phase (Table 2), 82.7% of all Phase 1 clinical trials were operated in North America (52.8%) and Europe (29.9%), whereas less than 3.0% were conducted in Latin America and Africa. Compared to Phase 1 clinical trials, the proportion of Phase 2 and Phase 3 clinical trials decreased in North America from 54.4% to 36.0% and 17.6, respectively, while increasing in other regions. Over the two decades, North America and Europe experienced the highest increase in the number of ADRD clinical trials, with little to no change in Latin America and Africa (Figure 2, Panel B). The average clinical trial density by regions, which accounts for people living with dementia (per million), is shown in Figure 3. North America experienced the largest increase in trial density over the last two decades (1.9 to 14.0), followed by Europe from 0.1 to 11.7, whereas Asia-Pacific (0.3 to 1.4), Africa (0 to 0.8), and Latin America (0 to 1.7) experience little growth in trial density (Table S2). At a country level, 21.5% of all clinical trials were conducted in the United States, followed by the United Kingdom and Canada (5.7%), Germany (5.1%), and France (4.5%).

#### 3.1.3 | Clinical trials by funding source

Distribution of funding sources by region are shown in Table S3. In summary, industry was the major funder for ADRD clinical trials, providing funds for 81.4% of all clinical trials. Research/academia centers, Public-Private partnerships (including government, industry and/or non-profit), and government provided funding for 8.6%, 5.4%, and 4.6% of all ADRD clinical trials, respectively.

#### 3.1.4 | Barriers to conducting dementia clinical trials in LMICs

Eighteen studies were included in the review to identify barriers to clinical trial implementation in LMICs (Panel 1). Fifteen of the included studies assessed barriers of conducting research studies on nonspecific health conditions; the remaining studies assessed barriers to conducting clinical trials. Among the 18 articles included, six studies used qualitative methods such as focus group discussion and individual interviews, and 13 articles used quantitative methods. In addition, to the systematic review, data from 19 qualitative semi-structured interviews were used to supplement literature findings. The main barriers to conducting clinical trials in developing countries are described below.

#### 3.1.5 | Resource limitations

Limited research funding was consistently one the most relevant barriers for research and clinical trial implementation.^28–34^ Despite, some variability across studies according to region and country, ≈45% to ≈80% of researches report limited funding for research implementation.^28–34^ Low remuneration, under-resourced research centers, and a lack of access to research and laboratory materials were also highlighted in several studies.^28,31,34^ In addition, during the 1:1 qualitative semi-structured interviews, several experts highlighted little to no pharmaceutical-sponsored research and clinical trial activity. Researchers shared that LMICs are “not as profitable as countries in the global north.” LMICs cannot compete to satisfy pharmaceutical companies’ commercial drivers, as dementia often lacks government funding and prioritization within the health priorities. With limited potential for sales revenue, pharmaceutical participants reported that LMICs have fewer opportunities to participate in dementia clinical trials, since pharmaceutical companies aim to conduct research in countries where the data generated can be leveraged for regulatory purposes as they aim to sell their prospective new therapy.

#### 3.1.6 | Regulatory system obstacles

Lengthy turnaround times for regulatory approvals combined with complex and unclear guidelines were mentioned in most articles.^30–33,35,36^ Several reports highlighted that regulatory review timelines were disruptive of grant timelines, leading to not meeting recruitment goals or grants to expire.^35,36^ Limited regulatory and subject-matter experience combined with limited ethical review capacity were also reported.^30,33^ These limitations were acknowledged during the 1:1 qualitative semi-structured interview across multiple disciplines. Pharmaceutical leaders and contract research organizations (CROs) positioned lengthy and unclear regulatory approvals as relevant limitations for CT implementation. Both, pharmaceutical leaders and CROs consider that such limitation should be solve by local regulatory agencies, government, and researchers.

#### 3.1.7 | Operational barriers

Unsupportive administrative system(s) and/or lack of skilled administrative personnel were mentioned in most articles.^30–34,36,37^In addition, other reports included lengthy and complex administrative environment, institutional barriers to obtaining and executing grants, understaffed administrative teams, and no coordinated approach to research between academics and administrative personnel. These limitations influence trial site development and investment. Pharmaceutical participants in the 1:1 qualitative semi-structured interview highlighted that, because of the amount of financial and scientific risk involved with ADRD clinical trials, pharmaceutical companies will often work with established partners, including former colleagues and their referral networks, or clinical trial sites in nearby countries with similar cultural norms and language.

#### 3.1.8 | Population and individual level

Several factors at a population and individual level were reported to impact trial implementation. At an individual level there is limited access to research training and exposure during early career years.^28–31,37,38^ In addition, limited access to leadership and mentorship was highlighted as a limiting factor to engage in research.^29,30,34,38^ Some studies reported absence of suitable research infrastructure leading to the lack of scientific atmosphere (e.g., lack of collaboration, poor communication among stakeholders, and low remuneration for research activities).^32,34,38^ At a population level, there is little awareness of clinical research needs and limited understanding of research participation.^32,37^ All participants included in 1:1 qualitative semi-structured interviews highlighted that developing working relationships for ADRD clinical trials is complex and reported lack of trust on their clinical trial partners, particularly in the relationships between for-profit and public organizations.

### 3.2 | A regional perspective on clinical trial implementation in LMICs

#### 3.2.1 | Latin America

Countries in Latin America (LatAm) and the global Latino community will experience the largest dementia increase compared to other ethnic groups.^2,39^ Although research is only beginning to uncover the risk and impact of ADRD among Latinos, what we have learned to date suggests that Latinos have a 1.5- to 2-fold greater risk of developing dementia than non Hispanic whites.^40^ Furthermore, within Latino populations, the picture is not so clear, with some Latinos subgroups having a higher risk of Alzheimer’s disease than others (e.g., Caribbean vs Mexican).^9,41^ The reason for these disparities among Hispanic sub-groups is still poorly understood and highly controversial, but differences in social determinants of health might also affect clinical trial recruitment and outcomes.^42^ One significant limitation of previous research is considering Latinos as a monolithic group. Latin America (or LatAm) is a diverse region with significant differences in admixture proportions^43,44^ and genetic risk factors across populations.^45–47^ The region is also diverse in terms of socioeconomic status, general health, comorbidities, nutrition, educational levels, and rural vs urban areas. These differences lead to heterogeneous prevalence rates of cognitive impairment and dementia.^41,48,49^ Access to health care and early diagnosis are also unresolved issues in the region, which may impact early recruitment and retention in clinical trials.^2^ To date, only 4.5% of ADRD clinical trials are conducted in LatAm and the overwhelming majority are concentrated in urban centers in Brazil, Argentina, Chile, Mexico, and Colombia, failing to reflect the true diversity of the region.^50,51^ Ongoing regional clinical trial platforms are now taking into consideration such differences and helping to implement regional clinical trials.^52,53^

#### 3.2.2 | Africa

By the year 2050, 212 million persons 60 years of age or older will be residing in the African continent.^54^ Similar to other LMICs regions, there is currently a dearth of clinical trials relating to ADRD in Africa. Relative to other regions, there are significant gaps in how ADRD impacts the 54 countries and over 3000 diverse ethnic groups in the African continent. Critical research areas to develop prior to clinical trial implementation include: (1) population-based studies on prevalence, incidence, and risk factors; (2) better characterization of the population in terms of genetic diversity and AD-related biomarkers; (3) validation of robust and culturally -sensitive cognitive assessments tools; and (4) capacity building and infrastructure development.^55^ In addition, poor mental health–seeking behavior, stigma, and low awareness are particularly relevant in the African region, which may limit clinical trial recruitment. Several communities, including caregivers, consider dementia as a feature of normal aging.^56^ Finally, a key element for the success of clinical trial implementation will be the creation of training programs aimed at preparing a new generation of ADRD specialists in the region. The African Dementia Consortium (AfDC), a platform for cooperation among dementia researchers and advocates across African countries, is now taking critical steps toward enhancing dementia research in the region.^55^

#### 3.2.3 | Asia-Pacific

The Asia-Pacific region represents countries that contain more than half of the world’s population, and the number of people living with dementia is estimated to increase from 23 million in 2015 to 71 million by the year 2050.^57^ However, evidence for the benefits of treatment for dementia is lacking and there is a need to systematically conduct clinical trials specific to these countries. Several sociodemographic features of Asian countries impact trial design and outcome measures in clinical trials. India in particular is characterized by cultural and linguistic diversity, high rates of illiteracy, and educational heterogeneity.^58,59^ A lack of awareness, stigma, and late diagnosis pose major challenges toward recruitment of patients at an early stage of the disease, and this gap is being addressed by advocacy measures at the levels of community and policy.^60^ Lower life-expectancy in India, high burden of vascular risk factors, and a diversity of genetic factors also affect clinical outcomes, and these factors should be systematically incorporated into clinical trial design.^61^ Efforts are ongoing to support clinical trial initiatives in Asia, and recruitment is rapidly increasing due to the development of infrastructural capability, lower trial costs, and a large numbers of patients.^62^

## 4 | DISCUSSION

In this study, we provide evidence on the limited number of ADRD-DMTs and barriers facing clinical trial implementation in LMICs. Developing countries will bear the greatest burden of ADRD by mid-century; however, only a fraction of the ADRD clinical trials are conducted in LMICs and there is significant under-representation of LMICs in historical and contemporary clinical trial platforms. Currently, less than 15% of ADRD clinical trials are being conducted in developing countries, which is a significant failure that inherently excludes the diversity of the population at risk for ADRD, highlighting the pressing need for clinical trial globalization. The comprehensive and mechanistic infrastructure in the development and implementation of clinical trials is severely lacking in LMICs. We identified several barriers to implementing ADRD trials globally including resource and economic limitations, poor efficiency of regulatory systems, unresolved operational complexities, and challenges with recruitment and retention.

Concentration of ADRD in HICs and the global north overlooks possible biological, ethnic, cultural, and socioeconomic heterogeneity that very likely influences treatment response and safety. Therefore, LMICs will need to pursue their own evidence-based clinical trial efficacy and safety, instead of adopting results from HIC research and generalizing them to their population.

Our study discovered several key economic and resource limitations underpinning global clinical trial implementation. Pharmaceutical companies and industry are the potential major investor in clinical trial development, investment, and partnerships with LMICs. However, by nature of being codified as LMICs, these countries are not viewed as lucrative as their well-invested HIC counterparts.^63,64^ Unfortunately, the commercial drivers that heavily influence ADRD clinical trials are directing pharmaceutical companies and industry investment to countries with greater financial resources and with a wider capacity to purchase the “end product” after successful trials.

It is also relevant to highlight that local governments in LMICs provide limited national budget allocation to research and development.^64^ The LMIC research environment is characterized by restricted access to research grants, undersized infrastructure, scarce equipment, and limited supply of reagents, which deter engagement and motivation toward research. LMIC universities redirect and concentrate efforts toward teaching, whereas the research support structure is limited or simply does not exist. As a result, researchers must devote significant time and effort to administration. Based on our findings, the lack of support from policymakers and stakeholders combined with the need for the development of a more research-oriented and supportive leadership/environment exerts a significant toll on research. These factors contribute to diminished scientific productivity and innovation,^64^ not from the shortfall of creativity from LMICs researchers but rather from an unacknowledged and unsupportive research environment.

A complex, inefficient, and usually lengthy regulatory system also hampers research and clinical trial development in LMICs. These obstacles serve as a deterrent to the collaborative partnership between pharmaceutical industries, non-profits organizations, and researchers engaging in ADRD research. A key driver for trial development is a rapid and efficient implementation, which may reduce trial duration and move toward key decision points that enable the maker of an ADRD therapy to seek regulatory approval to go to market. Finally, as identified by our study and highlighted by previous research,^29,30,34,38^ limited capacity building in areas of scientific research is a major limitation for trial implementation. Typically, academic institutions do not offer the resources for research or fellowships, or do not include research-oriented educational programs or are not included as part of the training curriculums.^64^

To help address challenges in ADRD global trial implementation, especially in LMICs, each region/country should develop a clear pathway toward addressing identified limitations. Although strategies should be tailored to the specific conditions of the region/country and thorough understanding of the local context, here we provide and discuss expert consensus recommendations to improve ADRD trial global representation (Panel 1). Key elements for ADRD clinical trial development include increasing research funding and infrastructure support through strategic partnerships between HICs and LMICs, strengthening regulatory response capacity and human research capital development, and building trust and awareness in the population. Global collaboration among HICs and LMICs is essential to foster clinical trial research. LMICs and HICs should work together to run ADRD trials that address therapeutic and safety questions relevant to both environments. At the same time, successful partnerships should allow for LMICs to rigorously test research hypotheses relevant to their local context or adapted to that particular context. Several HIC-LMIC initiatives (Dominantly Inherited Alzheimer’s Network Trial Unit-LatAm initiative, the LatAm Fingers, Alzheimer’s Prevention Initiative-Colombia) have already proven the relevance of a successful partnership to improve recruitment and inclusion of diverse population in ADRC clinical trial and at the same time providing more opportunities for LMICs to engage in clinical trial research.

In the context of regulatory capacity, local governments and academic institutions will need to implement changes aimed at reducing approval timeline and speed regulatory processes. Limiting the regulatory bureaucracy may attract industry and CROs as a way to speed up research development. Of note, this suggestion is not intended to weaken regulatory requirements but rather to strengthen an efficient regulatory response that is compliant with the International Conference on Harmonization and protects the safety of individuals involved in a study.

Finally, significant investment is required in research infrastructure and research-based academic centers to build research and human capital capacity. Investment in training professionals and administrative capacity to run ADRC trials is essential for the success in increasing clinical trials in LMICs. Moreover, academic centers and local governments need to invest in sustained long-term research support beyond limited training by providing access to adequate research funding, well-equipped laboratories, and adequate remuneration for the participants who invest their time, bodies, and trust in this enterprise. At a population level, clinical trialists should also consider and address possible factors that might affect trial participation including lack of awareness and stigma around dementia, limited access to health care leading to ADRD underdiagnosis or late diagnosis, and limited knowledge or trust in science.^65–67^

### 4.1 | Strengths and limitations

The present study is subject to limitations that may affect the interpretation and generalizability of results. First, clinicaltrials.gov and the WHO-ICTRP were the main sources for CT identification, and the validity of these sources to identify trials in LMICs is not clear. Therefore, we recognize the potential for under-reporting of CT in LMICs. If LMICs do not regularly report ongoing clinical trials in clinicaltrials.gov and/or WHO-ICTRP, this would be an important limitation for clinical trial implementation in LMICs. Second, our study addressed only ADRD clinical trials using DMTs. Limitations and barriers for the implementation of DMT clinical trials are likely different from the ones faced by non-pharmaceutical interventions. Future studies should explore the use of non-pharmaceutical interventions in low-resource settings and provide tailored recommendations for implementation. Third, due to the limited number of references highlighting limitations to ADRD clinical trials implementation in LMICs, our study was not designed to exclude reports based on quality assessments, which might bias some of the interpretations. It is worth noting that our study explored only regional differences by economic development status without considering country-specific disparities that might be present in urban versus rural areas. These considerations are relevant for both LMICs and HICs, where clinical trial access can be particularly limited for those in rural regions or those in medically underserved communities.^68^ Factors limiting trial access to rural communities include limited transportation, awareness of trials, socioeconomic resources, ADRD underdiagnosis, and unequal distribution of trial sites.^69–71^

Despite these limitations, there are several strengths to the current approach. The findings presented here provide an overview of ADRD clinical trial distribution by region, country, and income level to further stimulate critical thinking and integrative discussion about barriers for clinical trial implementation on a global scale. More importantly, they highlight the persistent and systematic practices that yield a low number of ADRD trials in LMICs and the very small share of ADRD trials including populations from Latin America, Asia, and Africa. These findings provide new knowledge and recommendations for overcoming existing barriers for trial implementation, increasing diverse representation in ADRD clinical trials, and expanding access to LMICs. Future studies should explore rural versus urban access to clinical trials both in HICs and LMICs.

## Supplementary Material

Supplemental Material

## Figures and Tables

**Table 1:** Number of ADRD clinical trials by economic development status, 2000–2021.

	High Income (N = 77)	Upper-middle Income (N = 55)	Low and Middle Income (N = 82)
**Phase 1, n(%)**	393 (97.0)	10 (2.5)	2 (0.5)
**Phase 1/2, n(%)**	93 (92.1)	5 (5.0)	3 (3.0)
**Phase 2, n(%)**	975 (91.9)	69 (6.5)	17 (1.6)
**Phase 2/3, n(%)**	155 (90.6)	16 (9.4)	0 (0.0)
**Phase 3, n(%)**	1374 (83.2)	232 (14.1)	45 (2.7)
**Total, N** **^a^(%)**	3065 (88.4)	333 (9.6)	68 (2.0)

Abbreviations: DMT, disease-modifying therapy; ADRD, Alzheimer’s disease and related dementia.

a Refers to the number of clinical trials per country’s economic development status not to a unique DMT (same DMT could be in a clinical trial in more than one country/phase).

**Table 2:** Number of ADRD clinical trials per region, 2000–2021.

Phase, n(%)	Europe (N^b^ = 34)	North Americ^a^ (N^b^ = 2)	Asia-Pacific (N^b^ = 16)	Latin America (N^b^ = 11)	Africa (N^b^ = 3)
**Phase 1**	121(29.9)	214 (52.8)	62 (15.3)	4 (1.0)	4 (1.0)
**Phase 1/2**	33 (32.7)	45 (44.6)	22 (21.8)	1 (1.0)	0 (0.0)
**Phase 2**	510 (48.1)	374 (35.2)	130 (12.3)	26 (2.5)	21 (2.0)
**Phase 2/3**	84 (49.1)	44 (25.7)	25 (14.6)	15 (8.8)	3 (1.8)
**Phase 3**	958 (58.0)	271 (16.4)	271 (16.4)	109 (6.6)	42 (2.5)
**Total, N^c^(%)**	1781 (51.4)	950 (27.4)	510 (14.7)	155 (4.5)	70 (2.0)

Abbreviations: DMT, disease-modifying therapy; ADRD, Alzheimer's disease and related dementia.

a Includes Canada and United States only.

b Indicates the number of countries with at least one registered clinical trial in the region (not the total number of countries in the region).

c Refers to the number of clinical trials per region not to a unique DMT because the same DMT could be in a clinical trial in more than one country/phase.

**PANEL 1:** Barriers to conducting ADRD clinical trials and recommendations for trial implementation in LMICs.

Barriers	Limiting factors/areas in for improvement	Recommendations for trial implementation in LMICs
**Operational**	-Unsupportive administrative system^30–34,37^ -Lack of skilled administrative personnel^33,36^	-Strengthen institutional administration research capacity by increasing staff and training, in addition to addressing structures not functioning effectively. -Apply for research funding aimed at developing research institutional capacity to do randomized controlled trials (e.g., trial administration and management, ethics review processes, and engaging policy-makers). Support from HICs and non-profit organizations will be key to support such efforts. -Funders should allow funding allocation to train local administrative staff.
**Regulatory**	-Delay of approval decisions.^32,33,35,36^ -Complex and inefficient regulatory system without established procedures.^30–32,36^	-Implement regular assessment of review committees and trial regulatory capacity aimed at avoiding unnecessary bureaucracy.
**Resources**	-Lack of funding^28–34^ -Lack of infrastructure^28,31,34,36^ -Lack of research materials^29,30,38^ -Limited human development and expenditure in research education^28–31,36–38^	-Develop mutually beneficial partnerships between research institutions in highland low-income countries (the Fogarty International Centre of the US National Institutes of Health provides research grants for faculty in LMICs). -Research institutions in LMICs should implement systematic needs assessments (e.g., staffing levels, training, and infrastructure needs) to adapt to the changing research landscape. Assessment should take into account current capacity in place and plan for future capacity needs. -Identification of research gaps and opportunities should be followed by engagement with appropriate stakeholders. -At a national and regional level, the development of a clinical trial forum composed of local researchers, governmental representation, private sector, and international partners may help in setting a national priority to facilitate clinical trial implementation. -Foster greater cooperation and collaboration between funding agencies willing to support research initiatives in LMICs.
**Population**	-Lack of awareness^30,32,34,36,37^ -Lack of trust in science^36^	-Researchers must speak with communities and their representatives about the research goals and public health relevance for future treatments. Addressing the population concerns and beliefs about research/science is key for successful recruitment, enrollment, and retention.
**Individual**	-Lack of motivation^30,38^ -Need for leadership^29,30,34,38^ - Lack of interest by policy-makers^28,30,32,34^	-Identify young leaders (e.g., researchers, scientists, doctors, trialists) who are committed to research in clinical trials and support professional development. -Existing senior leaders should implement a structured mentoring regimen and a clear succession plan to facilitate the development of the younger generation. -Rely on institutional existing mentorship capacity to train a new generation of clinical trialist. -Establish regional and international experts as co-mentors and co-supervision aimed at creating a mutual understanding of local needs, processes, and clinical trials skills.

## Data Availability

Data supporting the results reported in this paper will be available immediately following publication indefinitely to anyone who wishes to access the data, for any purpose. Individuals who would like to access the data should contact the corresponding author.
