# Supplementary material for: A call for clinical trial globalization in Alzheimer’s disease and related dementia
Source: Alzheimers Dement. Author manuscript; Available in PMC 2023 Sep 28. (PMC10450094; doi:10.1002/alz.12995)
Supplement: Supplemental Material [file NIHMS1889822-supplement-Supplemental_Material.docx]

**Supplemental Material: A call for clinical trial globalization in Alzheimer Disease and Related Dementia.**

Jorge J Llibre-Guerra^1,2,3^, Anika Heavener^4^, Sonia Maria Dozzi Brucki^5^, Juan Pablo Díaz Marante^6^, Maritza Pintado-Caipa^7^, Yaohua Chen^8^, María Isabel Behrens^9^, Angela Hardi^10^, Arianna Admirall-Sanchez^11^, Rufus Akinyemi^12^, Survana Alladi^13^, Karen A. Dorsman^14^, Ana M Rodriguez-Salgado^15^, Joel Solorzano^16^, Ganesh M Babulal^1,3,17,18^, for the Diversity and Disparity Professional Interest Area Increasing Sustained Diversity in Clinical Trials Working Group *.

1. Department of Neurology, Washington University School of Medicine in St. Louis, USA
2. Dominantly Inherited Alzheimer’s Network Trial Unit
3. Institute of Public Health, Washington University in St. Louis, St. Louis, Missouri, USA
4. Harvard Medical School Department of Global Health and Social Medicine
5. Cognitive and Behavioral Neurology Unit, Department of Neurology, University of Sao Paulo, Brazil
6. Department of primary care, Espirutu Santo, Brazil
7. Instituto Peruano de Neurociencias, Peru. Atlantic Fellow for Equity in Brain Health of the Global Brain Health Institute, Peru
8. Lille Neurosciences & Cognition, Degenerative and Vascular Cognitive Disorders, Lille, France
9. Departamento de Neurología y Neurocirugía, Hospital Clínico Universidad de Chile, Independencia, Santiago, Chile.
10. Becker Medical Library, Washington University School of Medicine, St. Louis, USA.
11. Centre of Public Health, Trinity College Dublin, Ireland
12. Centre for Genomic and Precision Medicine, College of Medicine, UI, Ibadan, Nigeria
13. National Institute of Mental Health and Neuroscience, India
14. University of Texas Southwestern Medical Center, Dallas, Texas, USA
15. Global Brain Health Institute
16. Department of Medicine, Hospital Antonio Luaces Iralola
17. Department of Clinical Research and Leadership, The George Washington University School of Medicine and Health Sciences, Washington, DC, USA
18. Department of Psychology, University of Johannesburg, Johannesburg, South Africa

*The Working Group to Increasing Sustained Diversity in Clinical Trials is a joint initiative between the Alzheimer Association, the Center for the Study of Race, Ethnicity & Equity at Washington University in St. Louis and the Global Brain Health Institute.

**Suplemental Material 1: Increasing Sustained Diversity in Clinical Trials Working Group**

A.1-**Working Group** **Members:** Cristiano Aguzzoli, Rufus Akinyemi, Survana Alladi, Ganesh M Babulal, Mircea Balasa, Joyce Balls-Berry, Laura Booi, Maria Isabel Behrens, Sonia Brucki, Yaohua Chen, Mary Dalsin, Tselmen Daria, Shana Dodge, Maria Victoria Erbella, Arshad Faheem, Matthew Gabel, Ladan Ghazi Saidi, Rafi Hadad, Fasihah Irfani Fitri, Jorge J Llibre Guerra, Erika Mariana Longoria, Doris Molina Henry, Rayven-Nikkita Collins, Maira Okada de Oliveira, Chukwuanugo Ogbuagu, Maritza Pintado Caipa, Binita Rajbanshi, Devora Rose, Cristiano Schaffer Aguzzoli, Olga Sánchez-Soliño, Ana Luisa Sosa, Clara Vila-Castelar, Berenice Werle, Yared Zewde, Jennifer Zitser.

**A.2- Mission, Specific Aims and Goals: ﻿**The mission of the working group is to provide guidance and recommendations that enable and facilitate AD clinical trial participation of diverse communities. The specific aims are to:

1) Identify limitations to recruitment, enrollment, and retention.

2) Develop novel approaches that are more appealing/transparent to diverse communities.

3) Facilitate cross-program/initiative strategic communications and collaborations.

4) Leverage available evidence to develop strategies to address ethical complexities in recruitment.

5) Provide recommendations to clinical trial sites on recruitment.

The overarching goal of the WG is to engage broad segments of the public in Alzheimer’s and related dementias research, with a particular focus on underrepresented communities and Low- and middle-income countries (LMIC). The group will examine existing ethical frameworks and evidence-based methods to improve successful and ethical recruitment, consent, and retention of diverse communities into ADRD clinical trials.

**A.3-Collaborative approach:** Our working group (WG) aims to include academic and industry representatives, in addition to patients and advocacy groups. The WG will act as a liaison for multiple initiatives involved in increasing diversity in AD research and include representatives from diverse communities, High and Middle-Low Income Countries, clinical trialists, and other stakeholders. Throughout 2022 and 2024, all team members will meet remotely every 3 months to discuss and provide consensus recommendations for increasing diversity in clinical trials. The working group will utilize findings to develop approaches and address the identified challenges limiting diverse communities' participation in ADRD clinical trials. Successful interventions in AD will require both embrace of and critical engagement ethnoracially diverse individuals in AD research.

**Supplemental Material 2: Search Strategy: Research Barriers in LMIC Systematic Review Search Methods**

**Citation Library Stats**

Total number of results retrieved: 1207

Number of duplicates identified and removed: 397

Number of unique citations remaining: 810

**Search Strategies**

**Embase.com**

= 320 results on 3/2/2022

((research* OR ‘clinical trial*’ OR ‘clinical drug trial*’ OR ‘randomized controlled trial*’) NEAR/2 (barrier* OR obstacle* OR hurdle* OR impediment* OR constraint*)):ti,ab AND (('low income country'/exp OR 'middle income country'/exp OR 'Afghanistan'/exp OR 'Albania'/exp OR 'Algeria'/exp OR 'American Samoa'/exp OR 'Angola'/exp OR 'Argentina'/exp OR 'Armenia'/exp OR 'Azerbaijan'/exp OR 'Bangladesh'/exp OR 'Belarus'/exp OR ‘Belize’/exp OR ‘Benin’/exp OR ‘Bhutan’/exp OR ‘Bolivia’/exp OR 'Bosnia and Herzegovina'/exp OR 'Botswana'/exp OR ‘Brazil’/exp OR ‘Bulgaria’/exp OR ‘Burkina Faso’/exp OR ‘Burundi’/exp OR ‘Cabo Verde’/exp OR ‘Cambodia’/exp OR ‘Cameroon’/exp OR ‘Central African Republic’/exp OR ‘Chad’/exp OR ‘China’/exp OR ‘Colombia’/exp OR ‘Comoros’/exp OR 'Congo'/exp OR ‘Cost Rica’/exp OR 'Cote d`Ivoire'/exp OR ‘Cuba’/exp OR ‘Djibouti’/exp OR 'Democratic Republic Congo'/exp OR 'Dominica'/exp OR 'Dominican Republic'/exp OR ‘Ecuador’/exp OR 'Egypt'/exp OR 'El Salvador'/exp OR 'Equatorial Guinea'/exp OR 'Eritrea'/exp OR 'Eswatini'/exp OR 'Ethiopia'/exp OR 'Fiji'/exp OR ‘Gabon’/exp OR 'Gambia'/exp OR 'Georgia (republic)'/exp OR ‘Ghana’/exp OR ‘Grenada’/exp OR 'Guinea'/exp OR 'Guinea-Bissau'/exp OR ‘Guyana’/exp OR ‘Haiti’/exp OR ‘India’/exp OR ‘Indonesia’/exp OR 'Iran'/exp OR ‘Iraq’/exp OR ‘Jamaica’/exp OR ‘Jordan’/exp OR 'Kazakhstan'/exp OR ‘Kenya’/exp OR ‘Kiribati’/exp OR ‘Kosovo’/exp OR 'Kyrgyzstan'/exp OR 'Laos'/exp OR ‘Lebanon’/exp OR ‘Lesotho’/exp OR ‘Liberia’/exp OR ‘Libya’/exp OR ‘Madagascar’/exp OR ‘Malaysia’/exp OR ‘Maldives’/exp OR ‘Mali’/exp OR ‘Marshall Islands’/exp OR ‘Mauritania’/exp OR ‘Mauritius’/exp OR ‘Mexico’/exp OR 'Federated States of Micronesia'/exp OR ‘Moldova’/exp OR ‘Montenegro’/exp OR ‘Morocco’/exp OR ‘Mozambique’/exp OR ‘Myanmar’/exp OR ‘Namibia’/exp OR ‘Naura’/exp OR ‘Nepal’/exp OR ‘Nicaragua’/exp OR ‘Nigeria’/exp OR 'North Korea'/exp OR 'Republic of North Macedonia'/exp OR ‘Pakistan’/exp OR 'Papua New Guinea'/exp OR ‘Paraguay’/exp OR ‘Peru’/exp OR ‘Philippines’/exp OR ‘Romania’/exp OR 'Russian Federation'/exp OR 'Rwanda'/exp OR ‘Samoa’/exp OR 'Sao Tome and Principe'/exp OR 'Senegal'/exp OR 'Serbia'/exp OR 'Sierra Leone'/exp OR ‘Solomon Islands’/exp OR ‘Somalia’/exp OR ‘South Africa’/exp OR ‘South Sudan’/exp OR 'Sri Lanka'/exp OR 'Saint Lucia'/exp OR 'Saint Vincent and the Grenadines'/exp OR 'Sudan'/exp OR ‘Suriname’/exp OR 'Syrian Arab Republic'/exp OR ‘Tajikistan’/exp OR ‘Tanzania’/exp OR ‘Thailand’/exp OR 'Timor-Leste'/exp OR ‘Togo’/exp OR ‘Tonga’/exp OR ‘Tunisia’/exp OR ‘Turkey’/exp OR ‘Turkmenistan’/exp OR ‘Tuvalu’/exp OR ‘Uganda’/exp OR 'Ukraine'/exp OR ‘Uzbekistan’/exp OR ‘Vanuatu’/exp OR 'Venezuela'/exp OR ‘Vietnam’/exp OR 'Yemen'/exp OR ‘Zambia’/exp OR ‘Zimbabwe’/exp) OR (Afghan*OR Albania* OR Algeria* OR 'American samoa*' OR Angola* OR Armenia* OR Azerbaijan OR Azeri* OR Bangladesh* OR Belarus* OR Belize* OR Benin* OR Bhutan* OR Bolivia* OR Bosnia* OR Botswana OR Motswana OR Batswana OR Brazil* OR Bulgaria* OR Herzegovina OR 'Burkina Faso' OR Burkinabé* OR Burundi* OR 'Cabo Verde*' OR 'Cape Verde*' OR Cambodia* OR Cameroon* OR 'Central African’ OR Chad* OR China OR Chinese OR Columbia* OR Congo* OR ‘Comoros Eritrea*’ OR Comorian* OR 'Ivory Coast' OR 'Côte d Ivoire' OR Ivoirian OR ‘Costa Rica*’ OR Cuba* OR Djibouti OR Dominica* OR Ecuador* OR ‘Equitorial Guniea’ OR Equatoguinean* OR Egypt* OR 'El Salvador*' OR Ethiopia* OR Eritrea* OR Fiji* OR Gabon* OR Gambia* OR Ghana* OR Grenada OR Grenadian* OR Guatemala* OR Guinea* OR 'Guinea-Bissau' OR Guyan* OR Haiti* OR Honduras OR Honduran* OR India OR Indonesia* OR Iran* OR Iraq* OR Jamaica* OR Jordan* OR Kazakhstan* OR Kazakh* OR Kenya* OR Kiribati OR Kosovo OR Kosovar OR Kyrgyz OR Kyrgyzstan OR Lao OR Laos OR Laotian* OR Lebanon OR Lebanese OR Lesotho OR Liberia* OR Macedonian* OR Madagascar* OR Malawi* OR Malaysia* OR Maldives OR Maldivian* OR Mali* OR ‘Marshall Islands’ OR Marshallese OR Mauritania OR Mauritius OR Mauritian* OR Mexico OR Mexican* OR Micronesia* OR Moldova* OR Mongolia* OR Montenegro OR Montenegrin* OR Morocc* OR Mozambique* OR Mozambican* OR Myanmar OR Burma OR Namibia* OR Nauru* OR Nepal* OR Nicaragua* OR Nigeria* OR Niger* OR ‘North Korea*’ OR Pakistan* OR 'Papua New Guinea*' OR Paraguay* OR Peru* OR Perivian OR Philippines OR Filipino* OR Phillipino* OR ‘Republic of Georgia’ OR Romania* OR Russia* OR Rwanda* OR ‘Saint Lucia*' OR ‘Saint Vincent’ OR 'Sao Tome and Principe' OR Samoa* OR Senegal* OR Serbia* OR 'Sierra Leone*' OR 'Solomon Islands' OR Somali* OR ‘South Africa*’ OR ‘South Sudan' OR 'Sri Lanka*' OR Sudan* OR Suriname* OR Swaziland OR Swazi OR Syria* OR Tajikistan* OR Tanzania* OR Thai* OR 'Timor-Leste' OR Togo* OR Tonga* OR Tunisia* OR Turkey OR Turkish OR Turkmenistan OR Turkmen OR Tuvalu* OR Ukrain* OR Uganda* OR Uzbekistan* OR Uzbek* OR Vanuatu OR Venezuela* OR Vietnam* OR ‘Gaza strip’ OR Palestine OR ‘West Bank’ OR Yemen* OR Zambia* OR Zimbabwe* OR Zaire):ti,ab OR (((‘low income’ OR ‘low-income’ OR ‘lower income’) NEAR/2 (countr* OR nation* OR econom*)):ti,ab OR ((‘middle income’ OR ‘middle-income’) NEAR/2 (countr* OR nation* OR economy*)):ti,ab))

**Ovid-Medline All**

=254 results on 3/2/2022

((research* OR clinical trial* OR clinical drug trial* OR randomized controlled trial*) adj2 (barrier* OR obstacle* OR hurdle* OR impediment* OR constraint*)).ti,ab. AND ((Afghanistan/ OR Albania/ OR Algeria/ OR American Samoa/ OR Angola/ OR Argentina/ OR Armenia/ OR Azerbaijan/ OR Bangladesh/ OR Belarus/ OR Belize/ OR Benin/ OR Bhutan/ OR Bolivia/ OR "Bosnia and Herzegovina"/ OR Botswana/ OR Brazil/ OR Bulgaria/ OR Burkina Faso/ OR Burundi/ OR Cabo Verde/ OR Cambodia/ OR Cameroon/ OR Central African Republic/ OR Chad/ OR China/ OR Colombia/ OR Comoros/ OR Congo/ OR Costa Rica/ OR Cote d'Ivoire/ OR Cuba/ OR Djibouti/ OR "Democratic Republic of the Congo"/ OR Dominica/ OR Dominican Republic/ OR Ecuador/ OR Egypt/ OR El Salvador/ OR Equatorial Guinea/ OR Eritrea/ OR Eswatini/ OR Ethiopia/ OR Fiji/ OR Gabon/ OR Gambia/ OR "Georgia (Republic)"/ OR Ghana/ OR Grenada/ OR Guinea/ OR Guinea-Bissau/ OR Guyana/ OR Haiti/ OR India/ OR Indonesia/ OR Iran/ OR Iraq/ OR Jamaica/ OR Jordan/ OR Kazakhstan/ OR Kenya/ OR Kiribati/ OR Kosovo/ OR Kyrgyzstan/ OR Laos/ OR Lebanon/ OR Lesotho/ OR Liberia/ OR Libya/ OR Madagascar/ OR Malaysia/ OR Maldives/ OR Mali/ OR Micronesia/ OR Mauritania/ OR Mauritius/ OR Mexico/ OR Moldova/ OR Montenegro/ OR Morocco/ OR Mozambique/ OR Myanmar/ OR Namibia/ OR Naura/ OR Nepal/ OR Nicaragua/ OR Nigeria/ OR "Democratic People's Republic of Korea"/ OR "Republic of North Macedonia"/ OR Pakistan/ OR Papua New Guinea/ OR Paraguay/ OR Peru/ OR Philippines/ OR Romania/ OR exp Russia/ OR Rwanda/ OR Samoa/ OR Senegal/ OR Serbia/ OR Sierra Leone/ OR Melanesia/ OR Somalia/ OR South Africa/ OR South Sudan/ OR Sri Lanka/ OR Saint Lucia/ OR "Saint Vincent and the Grenadines"/ OR Sudan/ OR Suriname/ OR Syria/ OR Tajikistan/ OR Tanzania/ OR Thailand/ OR Timor-Leste/ OR Togo/ OR Tonga/ OR Tunisia/ OR Turkey/ OR Turkmenistan/ OR Uganda/ OR Ukraine/ OR Uzbekistan/ OR Vanuatu/ OR Venezuela/ OR Vietnam/ OR Yemen/ OR Zambia/ OR Zimbabwe/) OR (Afghan*OR Albania* OR Algeria* OR "American Samoa*" OR Angola* OR Armenia* OR Azerbaijan OR Azeri* OR Bangladesh* OR Belarus* OR Belize* OR Benin* OR Bhutan* OR Bolivia* OR Bosnia* OR Botswana OR Motswana OR Batswana OR Brazil* OR Bulgaria* OR Herzegovina OR "Burkina Faso" OR Burkinabe* OR Burundi* OR "Cabo Verde*" OR "Cape Verde*" OR Cambodia* OR Cameroon* OR "Central African" OR Chad* OR China OR Chinese OR Columbia* OR Congo* OR "Comoros Eritrea*" OR Comorian* OR "Ivory Coast" OR "Côte d Ivoire" OR Ivoirian OR "Costa Rica*" OR Cuba* OR Djibouti OR Dominica* OR Ecuador* OR "Equitorial Guniea*" OR Equatoguinean* OR Egypt* OR "El Salvador*" OR Ethiopia* OR Eritrea* OR Fiji* OR Gabon* OR Gambia* OR Ghana* OR Grenada OR Grenadian* OR Guatemala* OR Guinea* OR "Guinea-Bissau" OR Guyan* OR Haiti* OR Honduras OR Honduran* OR India OR Indonesia* OR Iran* OR Iraq* OR Jamaica* OR Jordan* OR Kazakhstan* OR Kazakh* OR Kenya* OR Kiribati OR Kosovo OR Kosovar OR Kyrgyz OR Kyrgyzstan OR Lao OR Laos OR Laotian* OR Lebanon OR Lebanese OR Lesotho OR Liberia* OR Macedonian* OR Madagascar* OR Malawi* OR Malaysia* OR Maldives OR Maldivian* OR Mali* OR "Marshall Islands" OR Marshallese OR Mauritania OR Mauritius OR Mauritian* OR Mexico OR Mexican* OR Micronesia* OR Moldova* OR Mongolia* OR Montenegro OR Montenegrin* OR Morocc* OR Mozambique* OR Mozambican* OR Myanmar OR Burma OR Namibia* OR Nauru* OR Nepal* OR Nicaragua* OR Nigeria* OR Niger* OR "North Korea*" OR Pakistan* OR "Papua New Guinea*" OR Paraguay* OR Peru* OR Perivian OR Philippines OR Filipino* OR Phillipino* OR "Republic of Georgia" OR Romania* OR Russia* OR Rwanda* OR "Saint Lucia*" OR "Saint Vincent" OR "Sao Tome and Principe" OR Samoa* OR Senegal* OR Serbia* OR "Sierra Leone*" OR "Solomon Islands" OR Somali* OR "South Africa*" OR "South Sudan" OR "Sri Lanka*" OR Sudan* OR Suriname* OR Swaziland OR Swazi OR Syria* OR Tajikistan* OR Tanzania* OR Thai* OR "Timor-Leste" OR Togo* OR Tonga* OR Tunisia* OR Turkey OR Turkish OR Turkmenistan OR Turkmen OR Tuvalu* OR Ukrain* OR Uganda* OR Uzbekistan* OR Uzbek* OR Vanuatu OR Venezuela* OR Vietnam* OR "Gaza strip" OR Palestine OR "West Bank" OR Yemen* OR Zambia* OR Zimbabwe* OR Zaire).ti,ab. OR ((("low income" OR "low-income" OR "lower income") adj2 (countr* OR nation* OR econom*)).ti,ab. OR (("middle income" OR "middle-income") adj2 (countr* OR nation* OR economy*)).ti,ab.))

**EBSCO Global Health**

=162 results on 3/2/2022

(AB((research* OR “clinical trial*” OR “clinical drug trial*” OR “randomized controlled trial*”) N2 (barrier* OR obstacle* OR hurdle* OR impediment* OR constraint*)) OR TI((research* OR “clinical trial*” OR “clinical drug trial*” OR “randomized controlled trial*”) N2 (barrier* OR obstacle* OR hurdle* OR impediment* OR constraint*))) AND ((AB[Afghan*] OR TI[Afghan*] OR AB[Albania*] OR TI[Albania*] OR AB[Algeria*] OR TI[Algeria*] OR AB["American Samoa*"] OR TI[“American Samoa*”] OR AB[Angola*] OR TI[Angola*] OR AB[Armenia*] OR TI[Armenia*] OR AB[Azerbaijan] OR TI[Azerbaijan] OR AB[Azeri*] OR TI[Azeri*] OR AB[Bangladesh*] OR TI[Bangladesh*] OR AB[Belarus*] OR TI[Belarus*] OR AB[Belize*] OR TI[Belize*] OR AB[Benin*] OR TI[Benin*] OR AB[Bhutan*] OR TI[Bhutan*] OR AB[Bolivia*] OR TI[Bolivia*] OR AB[Bosnia*] OR TI[Bosnia*] OR TI[Botswana] OR AB[Botswana] OR TI[Motswana] OR AB[Motswana] OR TI[Batswana] OR AB[Batswana] OR TI[Brazil*] OR AB[Brazil*] OR AB[Bulgaria*] OR TI[Bulgaria*] OR AB[Herzegovina] OR TI[Herzegovina] OR AB["Burkina Faso"] OR TI["Burkina Faso"] OR AB[Burkinabe*] OR TI[Burkinabe*] OR AB[Burundi*] OR TI[Burundi*] OR AB["Cabo Verde*"] OR TI["Cabo Verde*"] OR AB["Cape Verde*"] OR TI["Cape Verde*"] OR AB[Cambodia*] OR TI[Cambodia*] OR AB[Cameroon*] OR TI[Cameroon*] OR AB["Central African"] OR TI[“Central African”] OR AB[Chad*] OR TI[Chad*] OR AB[China] OR TI[China] OR AB[Chinese] OR TI[Chinese] OR AB[Columbia*] OR TI[Columbia*] OR AB[Congo*] OR TI[Congo*] OR AB["Comoros Eritrea*"] OR TI[“Comoros Eritrea*”] OR AB[Comorian*] OR TI[Comorian*] OR AB["Ivory Coast"] OR TI[“Ivory Coast”] OR AB["Côte d Ivoire"] OR TI["Côte d Ivoire"] OR AB[Ivoirian] OR TI[Ivoirian] OR AB["Costa Rica*"] OR TI[“Costa Rica*”] OR AB[Cuba*] OR TI[Cuba*] OR AB[Djibouti] OR TI[Djibouti] OR AB[Dominica*] OR TI[Dominica*] OR AB[Ecuador*] OR TI[Ecuador*] OR AB["Equitorial Guniea*"] OR TI["Equitorial Guniea*"] OR AB[Equatoguinean*] OR TI[Equatoguinean*] OR AB[Egypt*] OR TI[Egypt] OR AB["El Salvador*"] OR TI["El Salvador*"] OR AB[Ethiopia*] OR TI[Ethiopia*] OR AB[Eritrea*] OR TI[Eritrea*] OR AB[Fiji*] OR TI[Fiji*] OR AB[Gabon*] OR TI[Gabon*] OR AB[Gambia*] OR TI[Gambia*] OR AB[Ghana*] OR TI[Ghana*] OR AB[Grenada] OR TI[Grenada] OR AB[Grenadian*] OR TI[Grenadian*] OR AB[Guatemala*] OR TI[Guatemala*] OR AB[Guinea*] OR TI[Guinea*] OR AB["Guinea-Bissau"] OR TI["Guinea-Bissau"] OR AB[Guyan*] OR TI[Guyan*] OR AB[Haiti*] OR TI[Haiti*] OR AB[Honduras] OR TI[Honduras] OR AB[Honduran*] OR TI[Honduran*] OR AB[India] OR TI[India] OR AB[Indonesia*] OR TI[Indonesia*] OR AB[Iran*] OR TI[Iran*] OR AB[Iraq*] OR TI[Iraq*] OR AB[Jamaica*] OR TI[Jamaica*] OR AB[Jordan*] OR TI[Jordan*] OR AB[Kazakhstan*] OR TI[Kazakhstan*] OR AB[Kazakh*] OR TI[Kazakh*] OR AB[Kenya*] OR TI[Kenya*] OR AB[Kiribati] OR TI[Kiribati] OR AB[Kosovo] OR TI[Kosovo] OR AB[Kosovar] OR TI[Kosovar] OR AB[Kyrgyz] OR TI[Kyrgyz] OR AB[Kyrgyzstan] OR TI[Kyrgyzstan] OR AB[Lao] OR TI[Lao] OR AB[Laos] OR TI[Laos] OR AB[Laotian*] OR TI[Laotian*] OR AB[Lebanon] OR TI[Lebanon] OR AB[Lebanese] OR TI[Lebanese] OR AB[Lesotho] OR TI[Lesotho] OR AB[Liberia*] OR TI[Liberia*] OR AB[Macedonian*] OR TI[Macedonian*] OR AB[Madagascar*] OR TI[Madagascar*] OR AB[Malawi*] OR TI[Malawi*] OR AB[Malaysia*] OR TI[Malaysia*] OR AB[Maldives] OR TI[Maldives] OR AB[Maldivian*] OR TI[Maldivian*] OR AB[Mali*] OR TI[Mali*] OR AB["Marshall Islands"] OR TI[“Marshall Islands”] OR AB[Marshallese] TI[Marshallese] OR AB[Mauritania] OR TI[Mauritania] OR AB[Mauritius] OR TI[Mauritius] OR AB[Mauritian*] OR TI[Mauritian*] OR AB[Mexico] OR TI[Mexico] OR AB[Mexican*] OR TI[Mexican*] OR AB[Micronesia*] OR TI[Micronesia*] OR AB[Moldova*] OR TI[Moldova*] OR AB[Mongolia*] OR TI[Mongolia*] OR AB[Montenegro] OR TI[Montenegro] OR AB[Montenegrin*] OR TI[Montenegrin*] OR AB[Morocc*] OR TI[Morocc*] OR AB[Mozambique*] OR TI[Mozambique*] OR AB[Mozambican*] OR TI[Mozambican*] OR AB[Myanmar] OR TI[Myanmar] OR AB[Burma] OR TI[Burma] OR AB[Namibia*] OR TI[Namibia*] OR AB[Nauru*] OR TI[Nauru*] OR AB[Nepal*] OR TI[Nepal*] OR AB[Nicaragua*] OR TI[Nicaragua*] OR AB[Nigeria*] OR TI[Nigeria*] OR AB[Niger*] OR TI[Niger*] OR AB["North Korea*"] OR TI["North Korea*"] OR AB[Pakistan*] OR TI[Pakistan*] OR AB["Papua New Guinea*"] OR TI["Papua New Guinea*"] OR AB[Paraguay*] OR TI[Paraguay*] OR AB[Peru*] OR TI[Peru*] OR AB[Perivian] OR TI[Perivian] OR AB[Philippines] OR TI[Philippines] OR AB[Filipino*] OR TI[Filipino*] OR AB[Phillipino*] OR TI[Phillipino*] OR AB["Republic of Georgia"] OR TI[“Republic of Georgia”] OR AB[Romania*] OR TI[Romania*] OR AB[Russia*] OR TI[Russia*] OR AB[Rwanda*] OR TI[Rwanda*] OR AB["Saint Lucia*"] OR TI["Saint Lucia*"] OR AB["Saint Vincent"] OR TI["Saint Vincent"] OR AB["Sao Tome and Principe"] OR TI["Sao Tome and Principe"] OR AB[Samoa*] OR TI[Samoa*] OR AB[Senegal*] OR TI[Senegal*] OR AB[Serbia*] OR TI[Serbia*] OR AB["Sierra Leone*"] OR TI["Sierra Leone*"] OR AB["Solomon Islands"] OR TI["Solomon Islands"] OR AB[Somali*] OR TI[Somali*] OR AB["South Africa*"] OR TI["South Africa*"] OR AB["South Sudan"] OR TI["South Sudan"] OR AB["Sri Lanka*"] OR TI["Sri Lanka*"] OR AB[Sudan*] OR TI[Sudan*] OR AB[Suriname*] OR TI[Suriname*] OR AB[Swaziland] OR TI[Swaziland] OR AB[Swazi] OR TI[Swazi] OR AB[Syria*] OR TI[Syria*] OR AB[Tajikistan*] OR TI[Tajikistan*] OR AB[Tanzania*] OR TI[Tanzania*] OR AB[Thai*] OR TI[Thai*] OR AB["Timor-Leste"] OR TI["Timor-Leste"] OR AB[Togo*] OR TI[Togo] OR AB[Tonga*] OR TI[Tonga*] OR AB[Tunisia*] OR TI[Tunisia*] OR AB[Turkey] OR TI[Turkey] OR AB[Turkish] OR TI[Turkish] OR AB[Turkmenistan] OR TI[Turkmenistan] OR AB[Turkmen] OR TI[Turkmen] OR AB[Tuvalu*] OR TI[Tuvalu*] OR AB[Ukrain*] OR TI[Ukrain*] OR AB[Uganda*] OR TI[Uganda*] OR AB[Uzbekistan*] OR TI[Uzbekistan*] OR AB[Uzbek*] OR TI [Uzbek*] OR AB[Vanuatu] OR TI[Vanuatu] OR AB[Venezuela*] OR TI[Venezuela*] OR AB[Vietnam*] OR TI[Vietnam*] OR AB["Gaza strip"] OR TI["Gaza strip"] OR AB[Palestine] OR TI[Palestine] OR AB["West Bank"] OR TI["West Bank"] OR AB[Yemen*] OR TI[Yemen*] OR AB[Zambia*] OR TI[Zambia*] OR AB[Zimbabwe*] OR TI[Zimbabwe*] OR AB[Zaire] OR TI[Zaire]) OR (("low income" OR "low-income" OR "lower income") N2 (countr* OR nation* OR econom*)) OR (("middle income" OR "middle-income") N2 (countr* OR nation* OR economy*)))

**Scopus**

= 440 results on 3/2/2022; Books and Book chapters excluded from results

ITLE-ABS ( ( research* PRE/2 barrier* ) OR ( research* PRE/2 obstacle* ) OR ( research* PRE/2 hurdle* ) OR ( research* PRE/2 impediment* ) OR ( research PRE/2 constraint* ) OR ( "clinical trial*" PRE/2 barrier* ) OR ( "clinical trial*" PRE/2 obstacle* ) OR ( "clinical trial*" PRE/2 hurdle* ) OR ( "clinical trial*" PRE/2 impediment* ) OR ( "clinical trial*" PRE/2 constraint* ) OR ( "clinical drug trial*" PRE/2 barrier* ) OR ( "clinical drug trial*" PRE/2 obstacle* ) OR ( "clinical drug trial*" PRE/2 hurdle* ) OR ( "clinical drug trial*" PRE/2 impediment* ) OR ( "clinical drug trial*" PRE/2 constraint* ) OR ( "randomized controlled trial*" PRE/2 barrier* ) OR ( "randomized controlled trial*" PRE/2 obstacle* ) OR ( "randomized controlled trial*" PRE/2 hurdle* ) OR ( "randomized controlled trial*" PRE/2 impediment* ) OR ( "randomized controlled trial*" PRE/2 constraint* ) ) AND ( TITLE-ABS ( afghan* OR albania* OR algeria* OR "American Samoa*" OR angola* OR armenia* OR azerbaijan OR azeri* OR bangladesh* OR belarus* OR belize* OR benin* OR bhutan* OR bolivia* OR bosnia* OR botswana OR motswana OR batswana OR brazil* OR bulgaria* OR herzegovina OR "Burkina Faso" OR burkinabe* OR burundi* OR "Cabo Verde*" OR "Cape Verde*" OR cambodia* OR cameroon* OR "Central African" OR chad* OR china OR chinese OR columbia* OR congo* OR "Comoros Eritrea*" OR comorian* OR "Ivory Coast" OR "Côte d Ivoire" OR ivoirian OR "Costa Rica*" OR cuba* OR djibouti OR dominica* OR ecuador* OR "Equitorial Guniea*" OR equatoguinean* OR egypt* OR "El Salvador*" OR ethiopia* OR eritrea* OR fiji* OR gabon* OR gambia* OR ghana* OR grenada OR grenadian* OR guatemala* OR guinea* OR "Guinea-Bissau" OR guyan* OR haiti* OR honduras OR honduran* OR india OR indonesia* OR iran* OR iraq* OR jamaica* OR jordan* OR kazakhstan* OR kazakh* OR kenya* OR kiribati OR kosovo OR kosovar OR kyrgyz OR kyrgyzstan OR lao OR laos OR laotian* OR lebanon OR lebanese OR lesotho OR liberia* OR macedonian* OR madagascar* OR malawi* OR malaysia* OR maldives OR maldivian* OR mali* OR "Marshall Islands" OR marshallese OR mauritania OR mauritius OR mauritian* OR mexico OR mexican* OR micronesia* OR moldova* OR mongolia* OR montenegro OR montenegrin* OR morocc* OR mozambique* OR mozambican* OR myanmar OR burma OR namibia* OR nauru* OR nepal* OR nicaragua* OR nigeria* OR niger* OR "North Korea*" OR pakistan* OR "Papua New Guinea*" OR paraguay* OR peru* OR perivian OR philippines OR filipino* OR phillipino* OR "Republic of Georgia" OR romania* OR russia* OR rwanda* OR "Saint Lucia*" OR "Saint Vincent" OR "Sao Tome and Principe" OR samoa* OR senegal* OR serbia* OR "Sierra Leone*" OR "Solomon Islands" OR somali* OR "South Africa*" OR "South Sudan" OR "Sri Lanka*" OR sudan* OR suriname* OR swaziland OR swazi OR syria* OR tajikistan* OR tanzania* OR thai* OR "Timor-Leste" OR togo* OR tonga* OR tunisia* OR turkey OR turkish OR turkmenistan OR turkmen OR tuvalu* OR ukrain* OR uganda* OR uzbekistan* OR uzbek* OR vanuatu OR venezuela* OR vietnam* OR "Gaza strip" OR palestine OR "West Bank" OR yemen* OR zambia* OR zimbabwe* OR zaire ) ) OR ( TITLE-ABS-KEY ( ( "low income" W/2 countr* ) OR ( "low income" W/2 nation* ) OR ( "low income" W/2 econom* ) OR ( "low-income" W/2 countr* ) OR ( "low-income" W/2 nation* ) OR ( "low-income" W/2 econom* ) OR ( "lower income" W/2 countr* ) OR ( "lower income" W/2 nation* ) OR ( "lower income" W/2 econom* ) OR ( "middle income" W/2 countr* ) OR ( "middle income" W/2 nation* ) OR ( "middle income" W/2 econom* ) OR ( "middle-income" W/2 countr* ) OR ( "middle-income" W/2 nation* ) OR ( "middle-income" W/2 econom* ) ) ) AND ( EXCLUDE ( SRCTYPE , "k" ) OR EXCLUDE ( SRCTYPE , "b" ) )

**ProQuest Dissertations and Theses Global**

= 31 results on 3/3/2022 (Note: truncation removed from search terms due to limitation of the database)

TI(((research N/2 barrier) OR (research N/2 obstacle) OR (research N/2 hurdle) OR (research N/2 impediment) OR (research N/2 constraint) OR (“clinical trial” N/2 barrier) OR (“clinical trial” N/2 obstacle) OR (“clinical trial” N/2 hurdle) OR (“clinical trial” N/2 impediment) OR (“clinical trial” N/2 constraint) OR (“clinical drug trial” N/2 barrier) OR (“clinical drug trial” N/2 obstacle) OR (“clinical drug trial” N/2 hurdle) OR (“clinical drug trial” N/2 impediment) OR (“clinical drug trial” N/2 constraint) OR (“randomized controlled trial” N/2 barrier) OR (“randomized controlled trial” N/2 obstacle) OR (“randomized controlled trial” N/2 hurdle) OR (“randomized controlled trial” N/2 impediment) OR (“randomized controlled trial” N/2 constraint))) AND AB((("low income" N/2 countr*) OR ("low income” N/2 nation*) OR ("lower income" N/2 countr*) OR ("lower income" N/2 nation*) OR ("lower income" N/2 econom*) OR ("middle income" N/2 countr*) OR ("middle income" N/2 nation*) OR ("middle income" N/2 econom*) OR (afghanistan OR albania OR algeria OR "American Samoa" OR angola OR armenia OR azerbaijan OR azeri OR bangladesh OR belarus OR belize OR benin OR bhutan OR bolivia OR bosnia OR botswana OR motswana OR batswana OR brazil OR bulgaria OR herzegovina OR "Burkina Faso" OR burkinabe OR burundi OR "Cabo Verde" OR "Cape Verde" OR cambodia OR cameroon OR "Central African" OR chad OR china OR chinese OR columbia OR congo OR "Comoros Eritrea" OR comorian OR "Ivory Coast" OR "Cote d Ivoire" OR ivoirian OR "Costa Rica" OR cuba OR djibouti OR dominica OR ecuador OR "Equitorial Guniea" OR equatoguinean OR egypt OR "El Salvador" OR ethiopia OR eritrea OR fiji OR gabon OR gambia OR ghana OR grenada OR grenadian OR guatemala OR guinea OR "Guinea Bissau" OR guyan OR haiti OR honduras OR honduran OR india OR indonesia OR iran OR iraq OR jamaica OR jordan OR kazakhstan OR kazakh OR kenya OR kiribati OR kosovo OR kosovar OR kyrgyz OR kyrgyzstan OR lao OR laos OR laotian OR lebanon OR lebanese OR lesotho OR liberia OR macedonian OR madagascar OR malawi OR malaysia OR maldives OR maldivian OR mali OR "Marshall Islands" OR marshallese OR mauritania OR mauritius OR mauritian OR mexico OR mexican OR micronesia OR moldova OR mongolia OR montenegro OR montenegrin OR morocco OR mozambique OR mozambican OR myanmar OR burma OR namibia OR nauru OR nepal OR nicaragua OR nigeria OR niger OR "North Korea" OR pakistan OR "Papua New Guinea" OR paraguay OR peru OR peruvian OR philippines OR filipino OR phillipino OR "Republic of Georgia" OR romania OR russia OR rwanda OR "Saint Lucia" OR "Saint Vincent" OR "Sao Tome and Principe" OR samoa OR senegal OR serbia OR "Sierra Leone" OR "Solomon Islands" OR somali OR "South Africa" OR "South Sudan" OR "Sri Lanka" OR sudan OR suriname OR swaziland OR swazi OR syria OR tajikistan OR tanzania OR thai OR "Timor Leste" OR togo OR tonga OR tunisia OR turkey OR turkish OR turkmenistan OR turkmen OR tuvalu OR ukraine OR uganda OR uzbekistan OR uzbek OR vanuatu OR venezuela OR vietnam OR "Gaza strip" OR palestine OR "West Bank" OR yemen OR zambia OR zimbabwe OR zaire)))

**dementia**; Acute Confusional Senile Dementia; Aging; AIDS Dementia Complex; Alzheimer's Disease; Alzheimer Disease, Late Onset; AODR dementia; Blessed dementia rating scale; Brain Damage, Chronic; Breast discharge female; Cerebrovascular Disorders; Circumscribed atrophy of brain; Creutzfeldt-Jakob disease; Degenerative brain disorder; Dementia, Vascular; dementia; senile, Alzheimer's type (manifestation); Dementia acquired; Dementia aggravated; Dementia associated with another disease; Dementia due to Creutzfeldt Jakob disease; Dementia due to other general medical conditions; Dementia Due to Specified Medical Condition; Dementia in Alzheimer's disease, atypical or mixed type; Dementia with psychosis; Diffuse cerebral atrophy; Disorder brain (chronic); Drug-induced dementia; frontal dementia; HIV-1-Associated Cognitive Motor Complex; Lewy Body Disease; Mental deterioration; Mental Retardation; Multi-infarct dementia; Neurodegenerative Disorders; Other Alzheimer's disease; Pick Disease of the Brain; Post-Concussion Syndrome; Presenile dementia; Presenile dementia, Kraepelin type; Presenile dementia, uncomplicated; Presenile dementia with delirium; Presenile dementia with delusional features; Presenile dementia with depression; Prion Diseases; Pseudodementia; Secondary dementia; Semantic Dementia; Senile degeneration of brain, not elsewhere classified; senile dementia with delusional or depressive features; Severe intellectual disability; Toxic dementia

**Supplemental Table 1. Density of ADRD clinical trial by economic development status.**

|  | **High Income**  **(N=77)** | **Upper-middle Income**  **(N=55)** | **Low and middle Income**  **(N=82)** |
| --- | --- | --- | --- |
| **2000** | 1.1 | 0.0 | 0.0 |
| **2001** | 2.7 | 0.1 | 0.0 |
| **2002** | 1.9 | 0.2 | 0.0 |
| **2003** | 3.6 | 0.5 | 0.0 |
| **2004** | 3.5 | 0.2 | 0.0 |
| **2005** | 5.0 | 0.7 | 0.0 |
| **2006** | 8.1 | 0.6 | 0.4 |
| **2007** | 10.6 | 1.7 | 1.4 |
| **2008** | 12.0 | 2.1 | 0.7 |
| **2009** | 14.8 | 1.7 | 0.2 |
| **2010** | 7.1 | 1.1 | 1.3 |
| **2011** | 5.5 | 0.6 | 0.5 |
| **2012** | 10.6 | 1.1 | 0.6 |
| **2013** | 9.2 | 1.2 | 0.4 |
| **2014** | 12.7 | 1.7 | 0.5 |
| **2015** | 12.0 | 0.8 | 0.1 |
| **2016** | 13.4 | 1.1 | 0.4 |
| **2017** | 12.1 | 1.2 | 0.9 |
| **2018** | 9.5 | 1.0 | 0.6 |
| **2019** | 6.2 | 0.3 | 0.4 |
| **2020** | 11.3 | 0.5 | 0.3 |
| **2021** | 12.4 | 1.7 | 0.8 |

**Note:** Trial site year density was the number of registered clinical trial per years divided by regional dementia prevalence.

**Supplemental Table 2. Density of ADRD clinical trial by region.**

|  | **Europe** | **North America^a^** | **Asia-Pacific** | **Latin America** | **Africa** |
| --- | --- | --- | --- | --- | --- |
| **2000** | 0.1 | 1.9 | 0.3 | 0 | 0 |
| **2001** | 1.4 | 5.6 | 0.1 | 0 | 0 |
| **2002** | 1.2 | 2.6 | 0.3 | 0 | 0.7 |
| **2003** | 2.0 | 4.9 | 0.4 | 0 | 1.4 |
| **2004** | 2.4 | 5.8 | 0.1 | 0 | 1.4 |
| **2005** | 3.8 | 6.0 | 0.5 | 0 | 2.7 |
| **2006** | 8.0 | 7.3 | 0.7 | 0 | 1.9 |
| **2007** | 9.9 | 11.0 | 1.6 | 0 | 1.2 |
| **2008** | 12.3 | 9.1 | 1.7 | 3.2 | 3 |
| **2009** | 12.0 | 16.8 | 1.6 | 0 | 4.6 |
| **2010** | 6.9 | 8.1 | 1.0 | 2.5 | 0.6 |
| **2011** | 4.8 | 7.5 | 0.7 | 1 | 0.5 |
| **2012** | 9.9 | 10.1 | 1.4 | 4 | 3.1 |
| **2013** | 9.8 | 8.4 | 1.0 | 3.8 | 1.5 |
| **2014** | 13.2 | 10.8 | 1.8 | 3.4 | 2.4 |
| **2015** | 10.9 | 12.3 | 1.3 | 3.2 | 1.4 |
| **2016** | 13.0 | 12.1 | 1.9 | 0 | 0.9 |
| **2017** | 11.1 | 10.5 | 1.9 | 3 | 3.5 |
| **2018** | 8.3 | 11.2 | 1.5 | 2.1 | 0.4 |
| **2019** | 4.0 | 9.6 | 1.0 | 3.1 | 0.8 |
| **2020** | 10.4 | 10.9 | 1.5 | 2.6 | 0.8 |
| **2021** | 11.7 | 14.0 | 1.4 | 3.1 | 0.8 |

**Note:** Trial site year density was the number of registered clinical trial per year divided by regional dementia prevalence. ^a.^Includes USA and Canada, Mexico was included as part of Latin America.

**Supplemental Table 3. Clinical trial sponsors by region.**

|  | **Europe** | **North America^a^** | **Asia-Pacific** | **Latin America** | **Africa** |
| --- | --- | --- | --- | --- | --- |
| **Industry n(%)** | 1,587 (89.1) | 623 (65.6) | 418 (81.9) | 137 (88.4) | 59 (84.3) |
| **Public-Private^b^ n(%)** | 65 (3.6) | 79 (8.3) | 21 (4.1) | 10 (6.5) | 0 (0.0) |
| **Government n(%)** | 40 (2.2) | 113 (11.9) | 13 (2.5) | 0 (0.0) | 0 (0.0) |
| **Research/Academic n(%)** | 89 (5.0) | 135 (14.2) | 58 (11.5) | 8 (5.2) | 11 (15.7) |

^a.^Includes USA and Canada, Mexico was included as part of Latin America.^b.^ Includes Government, industry, and non-profits partnership.
